# Pre-existing resistance associated polymorphisms to NS3 protease inhibitors in treatment naïve HCV positive Pakistani patients

**DOI:** 10.1371/journal.pone.0231480

**Published:** 2020-04-10

**Authors:** Hafeez Ullah Khan, Sanaullah Khan, Muhammad Akbar Shah, Sobia Attaullah, Muhammad Arshad Malik

**Affiliations:** 1 Department of Zoology, University of Peshawar, Peshawar, Khyber Pakhtunkhwa, Pakistan; 2 Department of Medicine, Khyber Teaching Hospital, Peshawar, Khyber Pakhtunkhwa, Pakistan; 3 Department of Zoology, Islamia College Peshawar, Peshawar, Khyber Pakhtunkhwa, Pakistan; 4 Department of Biological Sciences, International Islamic University, Islamabad, Pakistan; University of Cincinnati College of Medicine, UNITED STATES

## Abstract

Chronic Hepatitis C Virus (HCV) infection is still a major health issue especially in endemic areas where fewer direct-acting virals (DAAs) are treatment options. Some HCV variants are associated with resistance and it reduces DAAs success where pre-existing variants prevail. In this study, we investigated resistance-associated polymorphisms (RAPs) in the HCV NS3 region from DAAs naïve Pakistani patients. 277 chronic HCV treatment naïve patients infected with genotype 1a, 3a and 3b were selected from various clinical centers in the capital city of Khyber Pakhtunkhwa province Pakistan. All the patients were included in this study after taking informed consent. HCV NS3 region was amplified and Sanger sequencing was performed to analyze RAPs to NS3 protease inhibitors. Of the total 29.24% (81/277) patients had detected with known RAPs *viz* V36A/G/L, T54S, V55A/D/I, Q80K/R, S122G/T/R, R155K/T/I, V158I, D168T/Q, and I170V. Among HCV-1a subjects overall RAPs found were 26.09% (12/46) and most prevalent substitutions were V36A/G (10.87%, 5/46) and R155K/T/I (8.70%, 4/46). Of the total HCV-3a infected patients, 30.95% were observed with RAPS. Ammon these, the most frequent substitutions were Q80R (13.69%, 23/168) followed by V36L (18.33%, 14/168) and V55I (5.95%, 10/168). Among HCV-3b patients, 26.98% were found with RAPs and S122R and Q80R were the dominant variants detected in 17.46 (11/63) and 12.70% (8/63) patients respectively. All these substitutions were associated with Boceprevir, Simeprevir, Telaprevir, and Paritaprevir. Single substitution in one sequence was found in 18.77% (52/277) and multiple in 10.46% (29/277). More than one RAP was frequent in HCV-3a sequences. Natural RAPs are common in chronic HCV patients infected with genotype 1a, 3a and 3b, the most prevalent subtypes in Pakistan. High prevalence of HCV NS3 RAPs suggested a large scale study of the NS3 gene before the introduction of NS3 protease inhibitors in Pakistan.

## Introduction

The global burden of chronic Hepatitis C Virus (HCV) infection according to WHO is about 71 million and Pakistan adds ~10 million to this pool [1]. Standards of care to control HCV have been changed from conventional interferon-based therapies to direct-acting antivirals (DAAs). They inhibit NS3 protease very efficiently and enhance sustained virologic response (SVR) in comparison with other therapeutic agents. Despite the increased SVR, poor fidelity of viral polymerase and the high rate of virus replication also lead to variants in infected patients before treatment [2]. This type of virus variants may prevail in a population and the consequence is in pre-existing resistance to DAAs in the entire area.

HCV NS3 protease inhibitors like Boceprevir, Telaprevir, and Simeprevir can help in virus clearance but 5–10% of the patients are reported with therapy failure, mostly associated with virological factors [3–6]. Among these, nucleotide substitutions in the HCV genome resulted in resistant variants that have been already reported [7–10]. Such potent substitutions in NS3 protease of the infecting HCV genotypes were associated with decreased SVR or even NS3 Protease inhibitors therapy failure [2, 11]. In this context, the presence of RAPs in the HCV genome is an important issue that may negatively influence the current DAAs therapy response.

Various studies have uncovered the prevalence of RAPs to NS3 protease by analyzing HCV isolates of treatment naïve patients [4,7,10–14], patients treated with DAAs or sequences retrived from the nucleotide sequence databases [2,6,8,9]. However, pre-existing baseline information about RAPs in HCV in Pakistani patients has not been addressed. In Pakistan currently, DAAs like NS5A inhibitor (Daclatasvir and Velpatasvir) and NS5B inhibitors (Sofosbuvir) are in use and recently NS3 protease inhibitor like Simeprevir is introduced to combat HCV. It is known that the accumulation of resistant variants produced due to selective therapy pressure may decline the treatment success. Preliminary studies regarding pre-existing resistance variants have not been conducted in this area. The main objectiove of this study was to explore the presence of RAPs within the NS3 region of HCV-1a, HCV-3a, and HCV-3b in treatment naïve patients in an area of Pakistan.

## Materials and methods

### Study population and sampling

HCV RNA positive patients who did not receive antiviral therapy were included in this study. Serum samples were collected from different medical settings and private clinics in the capital city of Khyber Pakhtunkhwa province Pakistan between 2016 and 2018. Patients with HBV or HIV co-infection were excluded from this study. Informed consent (verbal from illiterate and written from educated) was obtained from every patient included in this study. Illetrate patients after their consent were interviewed for general information and data was collected on a prescribed questionnaire. Samples were brought in cold chain and processed in Molecular Biology and Virology Laboratory at the Department of Zoology University of Peshawar, Pakistan.

### Study approvals

The study protocols including consent (verbal/written) were approved by the Advanced Studies and Research Board and the Ethical Committee Centre of Biotechnology & Microbiology, University of Peshawar Khyber Pakhtunkhwa Pakistan.

### RNA isolation, cDNA synthesis, and genotyping

RNA was isolated from 100μL serum sample using TRIzol^®^ LS Reagent (Invitrogen CA. USA) according to the supplier instructions. The first-strand cDNA of HCV RNA was synthesized using random hexamer and 200U/μL of RevertAid Reverse Transcriptase (ThermoFisher Scientific USA). cDNA of HCV core gene was processed for genotyping according to the protocol of Ohno et al. [15]. In brief, a fragment of the HCV core gene was amplified with nested PCR using 5U/μL of *Taq* DNA polymerase (ThermoFisher Scientific USA) in two aliquots different in primers specific to HCV genotypes. The amplified product was designated a specific genotype after electrophoresis in 2% agarose gel. Only HCV-1a, HCV-3a, and HCV-3b were included and the NS3 region was sequenced for genotype confirmation and mutation analyses.

### HCV NS3 gene amplification and sequencing

HCV NS3/4A regions of each genotype were amplified with nested PCR according to the protocol of Paolucci et al. [16]. The amplified product was cut from the gel and purified with QIAquick^®^ PCR Purification Kit (Qiagen USA) after electrophoresis. Sanger sequencing of the purified NS3 region was performed through ABI 3730XLs (Applied Biosystems, USA) by Macrogen Korea. The sequences obtained were submitted to GenBank under the accession numbers MN970192, MN970193, and MN970194.

### Sequence and data analysis

Nucleotide sequences were compared with reference sequences of GenBank accession numbers AF-009606 (HCV-1a), D17763 (HCV-3a) and D49374 (HCV-3b). All nucleotide sequences of HCV variants obtained were edited for analysis using BioEdit version 7.2 for windows. Sequence alignments were performed with MUSCLE online software. Mega-X software was used for nucleotide translation to amino acids and polymorphism identification. Data obtained was analyzed in percentages, standard deviation, and chi-square test through Statistix software version 10 for windows.

## Results

### Study population’s characteristics

HCV NS3 sequences were obtained of treatment naïve patients including 46 (16.60%) infected with HCV-1a, 168 (60.65%) with 3a and 63 (22.74%) with HCV-3b. Male to female ratio was 1.7 to 1 and the age range was 25–56 years. General characteristics like liver enzymes and virus quantification etc. of the study population are given in Table 1.

**Table 1:** General characteristics of the study population (N = 277).

Characteristics		Genotypes		
		1a [N = 46 (16.60%)]	3a [N = 168 (60.64%)]	3b [N = 63 (22.74%)]
Gender N (%)	M, 174 (62.81)	28 (60.86)	114 (67.85)	33 (52.38)
	F, 103 (37.18)	18 (39.13)	54 (32.14)	30 (47.61)
Age (years) Mean±SD		51.15±9.75	45.91±10.42	50.64±11.01
ALT, Mean±SD		58 ±5.32	56 ±3.05	56 ±3.43
AST Mean±SD		48±2.51	42±3.11	45±4.02
HCV Viral quantification Mean±SD		5.90±2.98	6.32±3.56	6.17±2.82

M = Male, F = Female, ALT = alanine aminotransferase, AST = aspartate aminotransferase

### Prevalence of RAPs in the NS3 region

**Fig 1:**
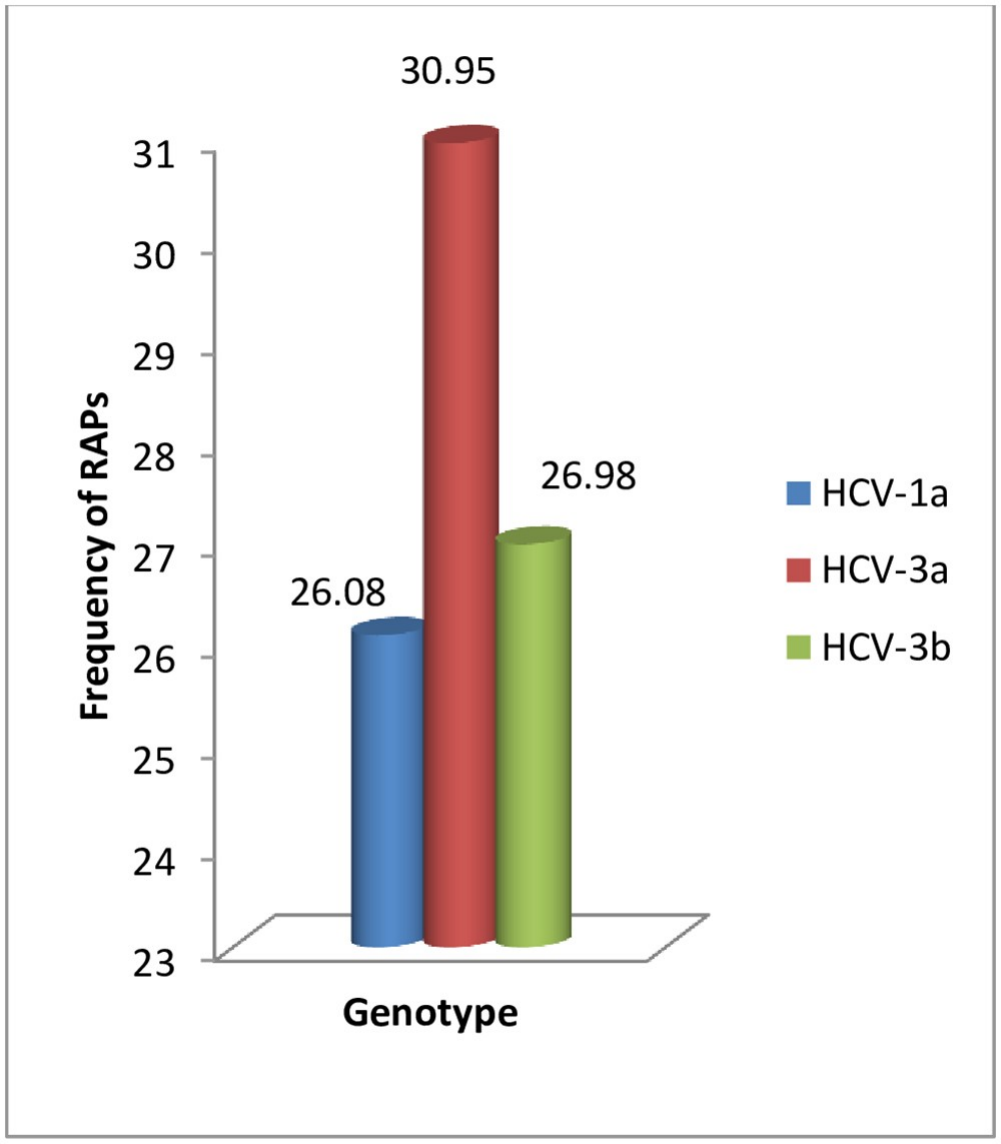
Frequency of RAPs among different HCV genotypes.

Sequence analysis of HCV NS3 region showed overall 29.24% (81/277) of infected patients harbored RAPS at positions 36, 54, 55, 80, 122, 155, 158, 168 and 170 (Table 2). Overall RAPs were found in 26.08% (12/46) of HCV-1a, 30.95% (52/168) of HCV-3a and 26.98% (17/63) of HCV-3b infected patients (Fig 1). The prevalence of RAPs was significantly higher (*P* = 0.026) in HCV-3a as compared to HCV-1a and HCV-3b. Among these, 64.19% (52/81) of individuals were found with one and 35.80% (29/81) with two RAPs.

**Table 2:** Pre-existing RAPs to NS3 protease inhibitors in different HCV genotypes.

Amino acid Position	HCV-1a (N = 46)	HCV-3a (N = 168)	HCV-3b (N = 63)
36	V36A/G (10.87%)	V36L (8.33%)	V36A/G (4.76%)
54	-	T54S (4.76%)	-
55	V55D/A (4.35%)	V55I (5.95%)	-
80	Q80K/R (6.52%)	Q80R (13.69%)	Q80K (12.70%)
122	S122G (2.17%)	-	S122T/R (17.46%)
155	R155K/T/I (8.70%)	-	
158	-	V158I (3.57%)	V158I (1.59%)
168	D168T (2.17%)	D168Q (2.38%)	-
170	-	I170V (4.17%)	I170V (3.17%)

Among HCV-1a sequences, five harbored V36A/G and two V55D/A, both these RAPs associated with resistance to Boceprevir, Telaprevir, and Paritaprevir. Three presented Q80K/R linked with Simeprevir, four R155K/T/I associated with resistance to Boceprevir and Telaprevir, while one each found with S122G associated with resistance to Simeprevir and D168T associated with resistance to Simeprevir and Paritaprevir (Table 2). Among these 17.39% (8/42) were found with one and 8.69% (4/42) with two RAPs. More than two RAPs in a single sequence of HCV-1a were not observed in this study.

Of the total HCV-3a infected patients, Q80R was the most prevalent RAP, associated with resistance to Simeprevir, followed by V36L/G linked with resistance to Boceprevir, Telaprevir and Paritaprevir. T54S associated with resistance to Boceprevir and Telaprevir, V55I associated with resistance to Paritaprevir, V158I linked with resistance to Boceprevir, D168Q associated with resistance to Simeprevir and Paritaprevir, and I170V linked with resistance to Boceprevir were also found (Table 2). In HCV-3a infected individuals single RAP was observed in 20.83% (35/168), two RAPs in 8.33% (14/168) and multiple in 1.79% (3/168).

In individuals infected with HCV-3b, S122T/R and Q80K were the most frequent RAPs identified. Both these RAPs were associated with resistance to Simeprevir. Resistant mutation V36A/G linked with resistance to Boceprevir, Telaprevir, and Paritaprevir were found in a few individuals. While V158I and I170V showed resistance to Boceprevir were also found in HCV-3b infected patients (Table 2). Single RAPs observed in nine (14.29%), duel in eight (12.70%) while no multiple mutations observed in this subtype.

At least one resistant mutation was observed in 18.77% (52/277) while more than two RAPs were observed in 10.46% (29/277) studied sequences. Multiple RAPs in a single sequence were less frequent and only found in 1.78% (3/168) of HCV-3a sequences. A considerable number of other substitutions not yet associated and reported with resistance were found.

The most common RAPs found among all the three genotypes were at position 36 [7.94% (22/277)] associated with resistance to Boceprevir, Telaprevir, and Paritaprevir and at position 80 [12.27% (34/277)] associated with resistance to Simeprevir.

## Discussion

The resistance of pathogens to medicines is continuously increasing and persistent viruses like HCV are still a challenge to antiviral therapies. DAAs provide significant and promising antiviral therapy to HCV but various host and viral factors and drug pressure against various genotypes do result in the emergence of resistant vial stains. Pre-existing polymorphism associated with resistance circulating in a geographic region should be considered as the main hurdle to specific DAAs’ success. HCV NS3 protease had been declared as a multifunctional therapy target with a high frequency of mutations.

Various studies conducted that elaborate HCV NS3 inhibitor resistance in genotype 1 and data regarding other genotypes are still limited to various geographical regions [17]. This is the first study from Pakistan that identified potent substitution to HCV NS3 protease in prevalent genotypes. HCV is endemic in Pakistan and prevalent genotypes in the study area and the entire region are 1a, 3a, and 3b [18,19]. DAAs therapy against HCV has been introduced a few years back but resistance mutations were not taken into consideration for therapy success. Pre-existing RAPs may influence DAAs therapy response and efficacy. We analyzed natural resistance-associated mutations to NS3 protease inhibitors in prevalent Pakistani HCV genotypes.

The overall prevalence of RAPs was higher in HCV-3a as compared to HCV-1a and HCV-3b and it may be due to sampling size or high prevalence of the genotypes in the study area. Among HCV-1a frequent substitutions observed during this study were V36A/G and R155K/T/I, in HCV-3a Q80R and V36L were abundant while among HCV-3b Q80K and S122R were prevalent. All these substitutions were associated with resistance to most of the presently licensed NS3 inhibitors like Simeprevir, Boceprevir, Telaprevir, and Paritaprevir [19–21]. Simeprevir response was studied in some countries and found low in various HCV variants with Q80K in patients infected with mono genotype [2,9,22,23]. Moreover, Simeprevir can increase the replication of HCV variants with Q80 and R155 substitutions even at a concentration of <1000nM [6]. Substitution at position 80 showed potential that promote the virus escape from NS3 protease inhibitors [24]. The substitution of Q80 was found in all the three HCV genotypes of the current study and deems failure of the available HCV NS3 inhibitors if introduced in the study area.

The range and occurrence of already detected mutations using deep sequencing procedures in individuals not treated with DAAs in chronic HCV have been explored in many countries. Some studies reported amino acid substitutions as R155K/T/Q, A156 S/T/V and I170A/T common in various regions [5, 25–27]. Various other mutations were detected generally did not confer resistance to known DAAs [28].

Mutation V55 was found in HCV-1a (4.35%) and HCV-3a (5.95%) and S122 in HCV1a (2.17%) and HCV-3b (17.46%) while R155 in HCV-1a (8.70%) only. These substitutions have a positive impact on viral replication and provide resistance to viral inhibitors [16]. But reduced susceptibility to Simeprevir due to substitution S122 was reported in HCV-1a and 1b while R155 reported with resistance to Boceprevir and Telaprevir [6,29,30].

There is no recommended standardized test available anywhere to identify RAPs before treatment, there is no consensus on such testingand it seems yet difficult to do [31]. In Pakistan, some DAAs like Sofosbuvir are available to treat HCV but resistance in a considerable number of patients is observed. So it is quite essential to advise and administer better DAA to circulating HCV genotypes variants. It is reported that HCV treated with a single NS3 inhibitor could result in variants with earlier drug resistance [32]. The combination of various DAAs with a different mode of action may reduce the chances of RAPs production, increased SVR and hence resistance [33]. In this study RAPs to more than one inhibitor were less common but not ignorable. This indicates that two or multiple RAPs will be an obstacle to the success of NS3 inhibitors in the study area.

## Conclusion

It is concluded that polymorphism within the NS3 region of HCV genotype 1a, 3a and 3b to various NS3 inhibitors in treatment naïve patients are prevalent in the study area. It is obvious that those HCV variants if circulated in the entire region, would escape from NS3 inhibitors.
